# Supplementary material for: OpenMM 7: Rapid development of high performance algorithms for molecular dynamics
Source: PLoS Comput Biol. 2017 Jul 26;13(7):e1005659. doi: 10.1371/journal.pcbi.1005659 (PMC5549999; doi:10.1371/journal.pcbi.1005659)
Supplement: S1 Source Code — (ZIP) [file pcbi.1005659.s002.zip › OpenMM-7.0.1-Source/docs/api-python/generated/simtk.openmm.app.topology.Topology.html]

Topology — OpenMM 7.0.1.dev-5e86c4f documentation


# Topology¶

*class* `simtk.openmm.app.topology.``Topology`¶
:   Topology stores the topological information about a system.

    The structure of a Topology object is similar to that of a PDB file. It consists of a set of Chains
    (often but not always corresponding to polymer chains). Each Chain contains a set of Residues,
    and each Residue contains a set of Atoms. In addition, the Topology stores a list of which atom
    pairs are bonded to each other, and the dimensions of the crystallographic unit cell.

    Atom and residue names should follow the PDB 3.0 nomenclature for all molecules for which one exists.

    `__init__`()¶
    :   Create a new Topology object

    Methods

    |  |  |
    | --- | --- |
    | `__init__`() | Create a new Topology object |
    | `addAtom`(name, element, residue[, id]) | Create a new Atom and add it to the Topology. |
    | `addBond`(atom1, atom2) | Create a new bond and add it to the Topology. |
    | `addChain`([id]) | Create a new Chain and add it to the Topology. |
    | `addResidue`(name, chain[, id]) | Create a new Residue and add it to the Topology. |
    | `atoms`() | Iterate over all Atoms in the Topology. |
    | `bonds`() | Iterate over all bonds (each represented as a tuple of two Atoms) in the Topology. |
    | `chains`() | Iterate over all Chains in the Topology. |
    | `createDisulfideBonds`(positions) | Identify disulfide bonds based on proximity and add them to the Topology. |
    | `createStandardBonds`() | Create bonds based on the atom and residue names for all standard residue types. |
    | `getNumAtoms`() | Return the number of atoms in the Topology. |
    | `getNumChains`() | Return the number of chains in the Topology. |
    | `getNumResidues`() | Return the number of residues in the Topology. |
    | `getPeriodicBoxVectors`() | Get the vectors defining the periodic box. |
    | `getUnitCellDimensions`() | Get the dimensions of the crystallographic unit cell. |
    | `loadBondDefinitions`(file) | Load an XML file containing definitions of bonds that should be used by createStandardBonds(). |
    | `residues`() | Iterate over all Residues in the Topology. |
    | `setPeriodicBoxVectors`(vectors) | Set the vectors defining the periodic box. |
    | `setUnitCellDimensions`(dimensions) | Set the dimensions of the crystallographic unit cell. |

    `getNumAtoms`()¶
    :   Return the number of atoms in the Topology.

    `getNumResidues`()¶
    :   Return the number of residues in the Topology.

    `getNumChains`()¶
    :   Return the number of chains in the Topology.

    `addChain`(*id=None*)¶
    :   Create a new Chain and add it to the Topology.

        |  |  |
        | --- | --- |
        | Parameters: | **id** (*string=None*) – An optional identifier for the chain. If this is omitted, an id is generated based on the chain index. |
        | Returns: | the newly created Chain |
        | Return type: | Chain |

    `addResidue`(*name*, *chain*, *id=None*)¶
    :   Create a new Residue and add it to the Topology.

        |  |  |
        | --- | --- |
        | Parameters: | - **name** (*string*) – The name of the residue to add - **chain** (*Chain*) – The Chain to add it to - **id** (*string=None*) – An optional identifier for the residue. If this is omitted, an id   is generated based on the residue index. |
        | Returns: | the newly created Residue |
        | Return type: | Residue |

    `addAtom`(*name*, *element*, *residue*, *id=None*)¶
    :   Create a new Atom and add it to the Topology.

        |  |  |
        | --- | --- |
        | Parameters: | - **name** (*string*) – The name of the atom to add - **element** (*Element*) – The element of the atom to add - **residue** (*Residue*) – The Residue to add it to - **id** (*string=None*) – An optional identifier for the atom. If this is omitted, an id is   generated based on the atom index. |
        | Returns: | the newly created Atom |
        | Return type: | Atom |

    `addBond`(*atom1*, *atom2*)¶
    :   Create a new bond and add it to the Topology.

        |  |  |
        | --- | --- |
        | Parameters: | - **atom1** (*Atom*) – The first Atom connected by the bond - **atom2** (*Atom*) – The second Atom connected by the bond |

    `chains`()¶
    :   Iterate over all Chains in the Topology.

    `residues`()¶
    :   Iterate over all Residues in the Topology.

    `atoms`()¶
    :   Iterate over all Atoms in the Topology.

    `bonds`()¶
    :   Iterate over all bonds (each represented as a tuple of two Atoms) in the Topology.

    `getPeriodicBoxVectors`()¶
    :   Get the vectors defining the periodic box.

        The return value may be None if this Topology does not represent a periodic structure.

    `setPeriodicBoxVectors`(*vectors*)¶
    :   Set the vectors defining the periodic box.

    `getUnitCellDimensions`()¶
    :   Get the dimensions of the crystallographic unit cell.

        The return value may be None if this Topology does not represent a periodic structure.

    `setUnitCellDimensions`(*dimensions*)¶
    :   Set the dimensions of the crystallographic unit cell.

        This method is an alternative to setPeriodicBoxVectors() for the case of a rectangular box. It sets
        the box vectors to be orthogonal to each other and to have the specified lengths.

    *static* `loadBondDefinitions`(*file*)¶
    :   Load an XML file containing definitions of bonds that should be used by createStandardBonds().

        The built in residues.xml file containing definitions for standard amino acids and nucleotides is loaded automatically.
        This method can be used to load additional definitions for other residue types. They will then be used in subsequent
        calls to createStandardBonds(). This is a static method, so it affects subsequent calls on all Topology objects.
        Also note that PDBFile calls createStandardBonds() automatically when a file is loaded, so the newly loaded definitions
        will be used for any PDB file loaded after this is called.

    `createStandardBonds`()¶
    :   Create bonds based on the atom and residue names for all standard residue types.

        Definitions for standard amino acids and nucleotides are built in. You can call loadBondDefinitions() to load
        additional definitions for other residue types.

    `createDisulfideBonds`(*positions*)¶
    :   Identify disulfide bonds based on proximity and add them to the
        Topology.

        |  |  |
        | --- | --- |
        | Parameters: | **positions** (*list*) – The list of atomic positions based on which to identify bonded atoms |

    `__delattr__`¶
    :   x.\_\_delattr\_\_(‘name’) <==> del x.name

    `__format__`()¶
    :   default object formatter

    `__getattribute__`¶
    :   x.\_\_getattribute\_\_(‘name’) <==> x.name

    `__hash__`¶

    `__reduce__`()¶
    :   helper for pickle

    `__reduce_ex__`()¶
    :   helper for pickle

    `__setattr__`¶
    :   x.\_\_setattr\_\_(‘name’, value) <==> x.name = value

    `__sizeof__`() → int¶
    :   size of object in memory, in bytes

    `__str__`¶

High performance molecular simulation on GPUs


### Navigation

- Application Layer
  - Loaders and Setup
  - Representation and Manipulation
  - Simulation
  - Reporting Output
  - Extras
- Library Layer

©2015, Stanford University and the Authors.
|
Powered by Sphinx 1.3.1
& Alabaster 0.7.7
|
Page source
